# A Manual of Midwifery

**Published:** 1912-06

**Authors:** 


### Reviews of Books.

**A Manual of Midwifery.** By BALFOUR MARSHALL, M.D., C.M. Pp. xviii, 401. Glasgow: James Maclehose & Sons. 1912.

This book is bound to become a popular one with students. It is written in a very easy, clear style, and at the same time it is methodical in its arrangement. All through the practical points are to the fore and theories are in the background. The systematic method of taking first the physiological or normal conditions, and then the pathological variations in the same subject, and describing what to expect in all cases on the one hand and what to be on the look-out for when difficulties arise on the other, is adopted. The actual ground covered in a book like this, taken from a course of lectures, is of course much the same as in most works of the kind; but the presentation of the same so as to make the subject easily grasped and retained is met with in but few, and in this book it is especially noticeable.

From the practitioner's point of view the work is particularly good in calling attention to just those little things which are useful to him by being a comfort to his patient.

The book is considerably lightened by the avoidance of long descriptions of uncommon complications, which the author recommends the reader to look up in larger text-books.

